# 超高效液相色谱-串联质谱法测定猪肉、鸡蛋、牛奶中9种食源性兴奋剂类药物残留

**DOI:** 10.3724/SP.J.1123.2021.04005

**Published:** 2022-02-08

**Authors:** Xuezhi LIU, Yinglian ZHAO, Yue MA, Shishi DONG, Bin WANG, Yang ZHANG

**Affiliations:** 1.中国检验检疫科学研究院综合检测中心, 北京 100123; 1. Chinese Academy of Inspection and Quarantine Comprehensive Test Center, Beijing 100123, China; 2.中检科(北京)测试技术有限公司, 北京 100176; 2. China Inspection Branch (Beijing) Test Technology Co., Ltd., Beijing 100176, China

**Keywords:** 超高效液相色谱-串联质谱, 食源性兴奋剂, 残留, 猪肉, 鸡蛋, 牛奶, ultra-performance liquid chromatography-tandem mass spectrometry (UPLC-MS/MS), food-borne stimulant drug, residues, pork, egg, milk

## Abstract

*β*-受体激动剂、*β*-阻断剂、蛋白同化制剂属于兴奋剂类药物,在动物饲养和屠宰过程中的违禁使用成为食源性兴奋剂类药物残留的来源,危害人类健康。目前*β*-受体激动剂、蛋白同化制剂的检测较多,*β*-阻断剂检测报道较少,动物源食品中*β*-阻断剂检测尚无标准方法。该文建立了超高效液相色谱-串联质谱测定猪肉、鸡蛋、牛奶中*β*-受体激动剂、*β*-阻断剂、蛋白同化制剂三大类共9种食源性兴奋剂类药物残留的方法。样品中加入乙酸铵缓冲液(pH 5.2)和*β*-葡萄糖醛酸酶/芳基硫酸酯酶,在37 ℃条件下酶解16 h,酶解后的试样冷却至室温,NaOH溶液调节pH 9.5后用乙腈提取,提取液盐析分层,经增强型脂质去除净化管(EMR-Lipid)净化,无水硫酸镁除水,氮吹浓缩至近干,残渣用1 mL乙腈-0.1%甲酸水溶液(1:9, v/v)溶解。以甲醇-0.1%甲酸水溶液作为流动相进行梯度洗脱,采用ACQUITY UPLC HSS T3色谱柱(100 mm×2.1 mm, 1.8 μm)分离,电喷雾正离子(ESI^+^)电离,多反应监测(MRM)模式下测定,基质匹配标准曲线内标法定量。实验优化了前处理过程中提取溶剂和pH对提取效率的影响,讨论了仪器分析过程中色谱柱、流动相、定容液等影响因素。结果表明:9种兴奋剂在0.5~20 μg/L浓度范围内线性关系良好,相关系数(*r*^2^)均大于0.99,方法检出限为0.3~0.6 μg/kg,定量限为1.0~2.0 μg/kg,在1、2、5倍定量限添加水平下,平均回收率为65.2%~117.0%,相对标准偏差(RSD)为1.3%~14.4%。应用建立的方法对市场上购入的猪肉、鸡蛋、牛奶三类动物源性食品进行测定,9种兴奋剂类药物残留均未检出。该方法快速、灵敏、准确、稳定,适用于猪肉、鸡蛋、牛奶等样品中9种食源性兴奋剂类药物残留的检测。

## Abstract (en)

*β*-Agonists, *β*-blockers, and protein assimilators are classified as stimulant drugs. Their illegal use during animal feeding and slaughtering leads to food-borne stimulant drug residues, which are harmful to human health. At present, methods for the detection of *β*-agonists and protein assimilators are prevalent, but those for the detection of *β*-blockers are rare. There is no national standard for the detection of *β*-blockers in food products of animal origin. A method based on ultra-performance liquid chromatography-tandem mass spectrometry (UPLC-MS/MS) was developed for the determination of nine food-borne stimulant drug residues, including *β*-agonists, *β*-blockers, and protein assimilators, in pork, egg, and milk. The optimal extraction conditions for this method were as follows: The samples were hydrolyzed with *β*-glucuronidase/aryl sulfate esterase in pH 5.2 ammonium acetate buffer. Enzymatic hydrolysis was carried out in a constant-temperature (37 ℃) water bath oscillator for 16 h. The enzymolyzed samples were cooled to room temperature and then extracted with acetonitrile, which was adjusted to pH 9.5 with NaOH solution. After extraction and homogeneous mixing, the extract was added to a salt package for salting out stratification. The clear supernatant was cleaned up using an enhanced lipid removal tube (EMR-lipid), which was pre-activated by water. Then, anhydrous magnesium sulfate was added to ensure dehydration of the extract and concentrated to near dryness under nitrogen flow. The residue was dissolved in 1 mL acetonitrile-0.1% formic acid aqueous solution (1:9, v/v). Separation was performed on an ACQUITY UPLC HSS T3 column (100 mm×2.1 mm, 1.8 μm) with gradient elution using methanol-0.1% formic acid aqueous solution as the mobile phase. The analytes were detected in the multiple reaction monitoring (MRM) mode after being ionized by an electrospray positive ion (ESI^+^). Quantitative analysis was performed by the internal standard method using matrix-matched calibration curves. The effects of the extraction solvent and pH on the extraction efficiency during pretreatment were optimized. The influence factors of different types of chromatographic column, mobile phase and dissolved solution in the process of instrumental analysis were discussed in detail. Under the optimal conditions, the method showed good linearity in the range of 0.5 to 20 μg/L, with correlation coefficients (*r*^2^) greater than 0.99. The limits of detection (LODs) and limits of quantification (LOQs) were in the range of 0.3-0.6 μg/kg and 1.0-2.0 μg/kg, respectively. The average recoveries of all the compounds ranged from 65.2% to 117.0% with relative standard deviations (RSDs) in range of 1.3%-14.4% at spiked levels of 1, 2, and 5 times the LOQs. The established method was used to determine the quality of animal-origin foods such as pork, eggs, and milk purchased from the market. The nine stimulant drug residues were not detected in these food samples. The analytical method is rapid, sensitive, accurate, and stable. It can be used for the determination of the nine food-borne stimulant drugs residue in pork, egg, and milk.

*β*-受体激动剂又称生长促进剂,俗称“瘦肉精”,家畜饲养时加大剂量长期使用,则有降低脂肪、提高瘦肉率、促进生长,改善肉质的作用。蛋白同化制剂又称同化激素,俗称合成类固醇,同样具有促进蛋白质合成,肌肉增生的作用。为了提高胴体品质、快速增重和提高饲料转化率,某些饲料生产商和家畜生产者违禁使用这些药物成为食源性兴奋剂类药物残留的来源。*β*-阻断剂,常用于运输前给动物注射以减轻动物的应激反应,防止因应激反应造成的肉品安全和质量大幅下降,且往往在屠宰前数小时使用,更容易造成药物残留,危害人类健康。农业部250号公告《食品动物中禁止使用的药物及其他化合物清单》包括*β*-兴奋剂类。世界反兴奋剂机构发布的《禁用清单国际标准》规定蛋白同化制剂、*β*-受体激动剂和*β*-阻断剂为禁止使用的兴奋剂类药物。建立多种兴奋剂的检测可满足食品安全应急事件处置的需要,以及防止重大赛事中运动员因食用被污染的食品而导致的兴奋剂阳性事件的出现。

食源性兴奋剂的检测有气相色谱-串联质谱法(GC-MS/MS)^[1]^、液相色谱-串联质谱法(LC-MS/MS)^[2,3,4,5,6,7]^、超高效液相色谱-四极杆/飞行时间质谱法(UPLC-Q-TOF/MS)^[8,9]^,其中GC-MS/MS需要衍生,操作繁琐;高分辨质谱仪多用于筛查与确证;LC-MS/MS测定时样品不用衍生化,灵敏度高、定性定量准确,在残留分析中应用最为广泛。我国现行有效的食源性兴奋剂检测标准^[10,11,12]^多以LC-MS/MS为主,其中*β*-受体激动剂和蛋白同化制剂的检测可参考相关标准,*β*-阻断剂尚无检测标准。

前处理方面,*β*-受体激动剂、蛋白同化制剂的测定多采用有机溶剂提取,固相萃取柱净化^[13,14,15,16]^,溶剂用量大,操作复杂。近年来,QuEChERS和Prime HLB因操作简便快速,溶剂用量少,与传统固相萃取柱相比无需活化和平衡,故在食源性兴奋剂检测中的应用越来越广泛,但大部分文献^[17,18,19,20,21,22]^都是针对单一种类兴奋剂的检测,且*β*-阻断剂检测报道较少。增强型脂质去除填料EMR(enhanced matrix removal)是一种改良的QuEChERS技术,该填料结合体积排阻和疏水作用两种机制,在不吸附目标化合物的同时捕获脂质等杂质,适用于脂肪、蛋白含量较高的动物源性食品中药物残留检测,齐鹤鸣等^[23]^采用EMR净化同时测定了*β*-受体激动剂和蛋白同化制剂两类兴奋剂,还未见EMR应用于食源性*β*-阻断剂检测的报道。

本文拟采用EMR净化、LC-MS/MS检测方法同时测定*β*-受体激动剂和阻断剂、蛋白同化制剂三大类共9种食源性兴奋剂,该方法操作简便,溶剂用量少,适用于大批量样品的快速测定。

## 1 实验部分

### 1.1 仪器、试剂与材料

ACQUITY UPLC TQ-S超高效液相色谱-串联质谱仪(美国Waters公司); Milli-Q超纯水仪(美国Millipore公司);离心机(德国Beckman公司);涡旋混合器(德国IKA公司)。

盐酸甲氧酪胺、阿替洛尔、酒石酸美托洛尔、克仑丙罗、脱氢表雄酮(纯度均大于97%,英国LGC公司);齐帕特罗、盐酸普萘洛尔、昔美酸沙美特罗、美替诺龙、氘代克伦特罗、氘代莱克多巴胺、氘代沙丁胺醇、氘代睾酮、氘代甲睾酮(质量浓度均为100 μg/mL,曼哈格检测技术有限公司)。

甲醇、乙腈(色谱纯,美国Fish公司);甲酸(色谱纯,北京百灵威科技有限公司);乙酸铵(色谱纯,美国Sigma公司);冰醋酸、氢氧化钠、无水硫酸钠、氯化钠、无水硫酸镁(分析纯,国药集团化学试剂有限公司); *β*-葡萄糖醛苷酸酶/芳基硫酸酯酶(含*β*-葡萄糖醛苷酸酶>100000 units/mL,芳基硫酸酯酶<20000 units/mL,上海安谱实验科技股份有限公司);增强型脂质去除净化管(EMR-Lipid dSPE)(美国Agilent公司)。

### 1.2 标准溶液的配制

分别准确称取适量(精确至0.01 mg)甲氧酪胺、阿替洛尔、美托洛尔、克仑丙罗、脱氢表雄酮于10 mL容量瓶中,用甲醇溶解并定容至刻度,配制成1.0 g/L的标准储备液,用甲醇稀释成100 mg/L的标准中间液。

分别准确量取适量(精确至0.01 mL)齐帕特罗、普萘洛尔、沙美特罗、美替诺龙及上述标准中间液用甲醇稀释成10 mg/L的混合标准中间液。

分别准确量取适量(精确至0.01 mL)氘代克伦特罗、氘代莱克多巴胺、氘代沙丁胺醇、氘代睾酮、氘代甲睾酮用甲醇稀释成10 mg/L的混合内标中间液。

称取6份空白基质样品,经样品前处理,作为空白基质溶液分别向其中加入250 μg/L混合内标工作液10 μL,加入不同体积的100 μg/L的混合外标工作液,配制成质量浓度为0.5、1.0、2.0、5.0、10.0、20.0 μg/L的空白基质标准溶液。

### 1.3 样品前处理

称取试样5.0 g(精确至0.01 g),放入50 mL离心管中,加入250 μg/L混合内标工作液40 μL,涡旋混匀,加入10 mL乙酸铵缓冲液(pH 5.2),加入50 μL *β*-葡萄糖醛酸酶/芳基硫酸酯酶,涡旋至混匀,于恒温水浴振荡器37 ℃振荡酶解16 h。酶解后的试样冷却至室温,10 mol/L氢氧化钠溶液调pH 9.5,加入12.0 mL乙腈涡旋提取2 min,加入盐包(4 g无水硫酸钠和1 g氯化钠)涡旋2 min,盐析分层,静置10 min后8000 r/min离心10 min。

移取6.0 mL乙腈提取上清液至EMR-Lipid净化管中(预先用3 mL水活化),涡旋2 min, 5000 r/min离心10 min,转移全部上清液于15 mL离心管中,加入反萃盐包(2 g无水硫酸镁),涡旋2 min, 5000 r/min离心5 min,取3.0 mL上清液于45 ℃氮吹至近干,加入1.0 mL乙腈-0.1%甲酸水溶液(1∶9, v/v)定容,涡旋混匀,过0.22 μm滤膜待测。

### 1.4 仪器条件

1.4.1 色谱条件

色谱柱:ACQUITY UPLC HSS T3(100 mm×2.1 mm, 1.8 μm);柱温:40 ℃;样品室温度:15 ℃;进样量:4 μL;流动相:A(甲醇)-B(0.1%甲酸水溶液);梯度洗脱程序:0~2.5 min, 0%A~20%A; 2.5~7 min, 20%A~95%A; 7~8 min, 95%A~0%A; 8~9 min, 0%A。

1.4.2 质谱条件

离子源:电喷雾电离源(ESI^+^);毛细管电压:3.5 kV;离子源温度:150 ℃;脱溶剂气温度:500 ℃;脱溶剂气流量:1000 L/h;碰撞气流量:0.15 mL/min;多反应监测模式(MRM)。9种兴奋剂的监测离子对、锥孔电压、碰撞能见表1, MRM图见图1。

**表 1:** 9种兴奋剂的质谱参数

Compound	t/min	Precursor (m/z)	Daughter (m/z)	Cone voltage/V	Collision energy/eV	Internal standard
3-O-Methyldopamine	2.35	168.1	119.1	30	16	salbutamol-D3
			151.1^*^	30	11	
Zilpaterol	2.90	262.1	185.2	30	22	ractopamine-D3
			244.3^*^	30	12	
Atenolol	2.98	267.2	145.1^*^	30	26	ractopamine-D3
			190.1	30	17	
Clenproperol	4.05	263.2	203.1^*^	30	16	ractopamine-D3
			132.2	30	27	
Metoprolol	4.55	268.3	74.1	30	20	clenbuterol-D9
			116.1^*^	30	17	
Propranolol	5.30	260.2	116.2	30	18	clenbuterol-D9
			183.2^*^	30	16	
Salmeterolxinafoate	6.19	416.4	380.3	30	15	clenbuterol-D9
			398.4^*^	30	12	
Dehydroepiandrosterone	7.06	271.2	213.1	25	20	testosterone-D3
			253.1^*^	30	15	
Metenolone	7.19	303.3	83.1	30	20	methyltestosterone-D3
			187.3^*^	30	20	
Salbutamol-D3	2.90	243.2	151.1	24	18	
Ractopamine-D3	4.15	305.2	167.0	30	18	
Clenbuterol-D9	4.40	286.1	204.1	30	16	
Testosterone-D3	6.98	292.4	97.1	30	24	
Methyltestosterone-D3	7.15	306.4	97.1	30	24	

* Quantitative ion.

**图1:**
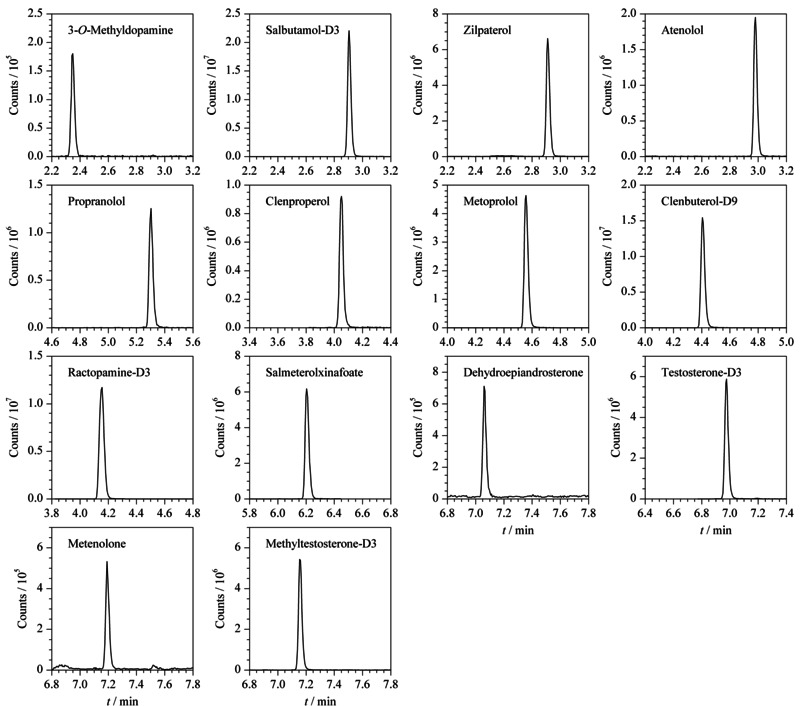
9种兴奋剂及5种内标的MRM色谱图(2.0 μg/L)

## 2 结果与讨论

### 2.1 前处理条件的选择

*β*-受体激动剂和蛋白同化制剂多为蛋白结合态存在,需要酸水解或酶解为游离态以利于有机溶剂的提取。实验采用*β*-葡萄糖醛苷酸酶/芳基硫酸酯酶进行酶解,考虑到除了蛋白同化制剂外,另外2类兴奋剂化合物结构中均含有氨基,故比较了酸性、中性和碱性提取条件对回收率的影响。由于猪肉、鸡蛋、牛奶基质中蛋白和脂肪含量较高,故提取液采用EMR进行净化。以阴性猪肉基质为例,实验比较了pH 5.2、pH 7.0、pH 8.0、pH 9.0、pH 10.0条件下分别用乙腈和乙酸乙酯作为提取溶剂的效果。结果表明,乙酸乙酯提取液经EMR净化时乳化明显,需较长时间高速离心破乳,且回收率差,其中齐帕特罗、克仑丙罗、普萘洛尔、美托洛尔的回收率在20%~50%之间,其他化合物均无回收。而乙腈提取并经EMR净化,回收率良好。比较了不同pH条件下乙腈提取的回收率和净化效果,发现大部分化合物的回收率随pH值增加而提高,齐帕特罗和阿替洛尔在pH 8.0以下回收率小于60%, pH 10.0时美托洛尔的回收率接近120%, pH对回收率的影响见图2。脱氢异雄酮在pH 8.0以下,提取效率高,但样品目标峰处干扰严重,影响定量与定性,pH增大提取效率略有下降,但净化效果明显,可能是由于pH 8.0以下提取目标物的同时提取了更多的杂质。为保证净化效果和满足回收率要求,选择在pH 9.5条件下提取,验证发现净化良好,回收率为65.2%~117.0%。综上所述,最终选择的提取条件和提取溶剂分别是pH 9.5和乙腈。

**图2:**
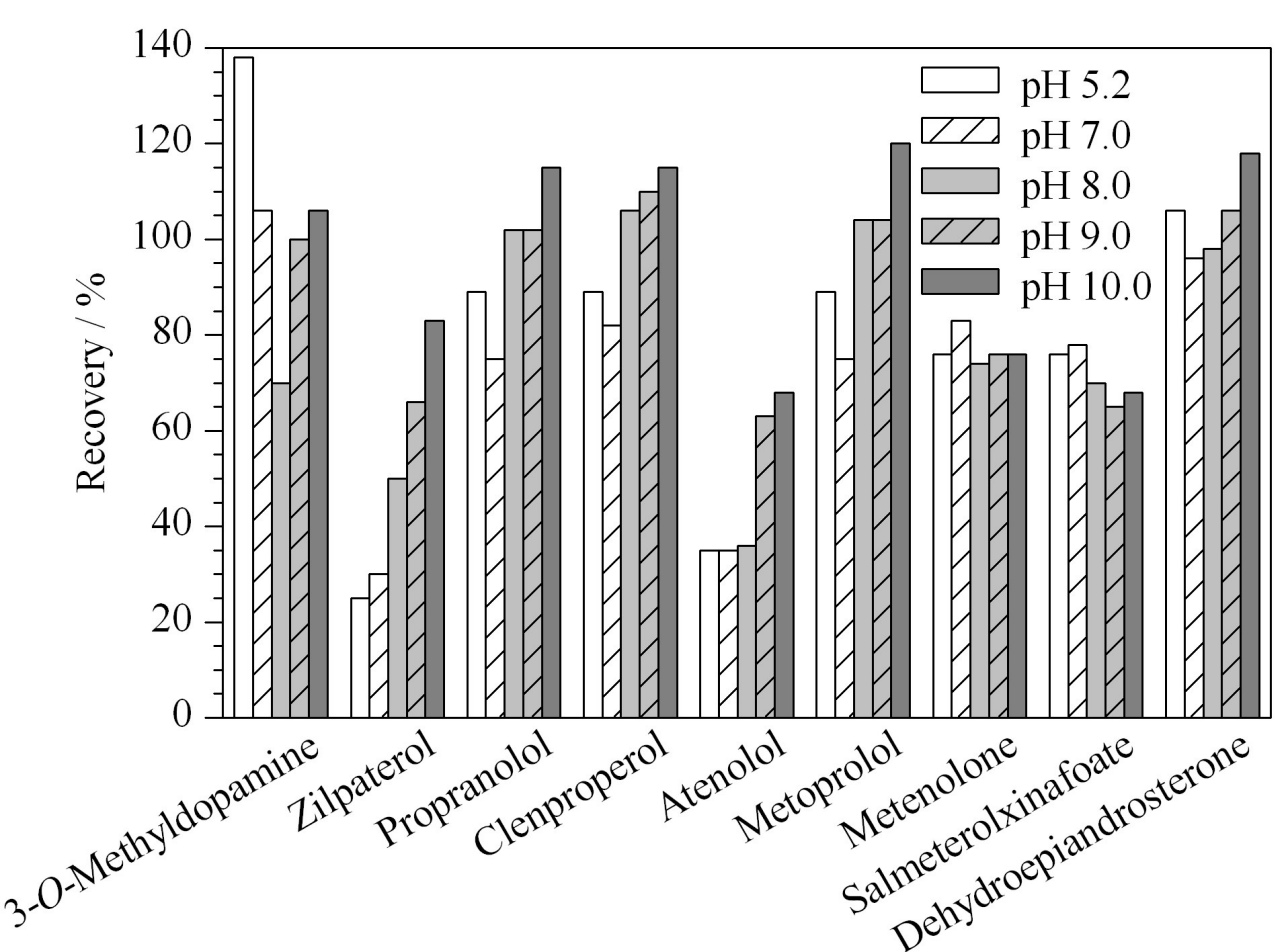
pH条件对9种兴奋剂回收率的影响

### 2.2 仪器条件的优化

2.2.1 色谱条件比较了BEH C18 (100 mm×2.1 mm, 1.7 μm)和HSS T3 (100 mm×2.1 mm, 1.8 μm)两种色谱柱,发现甲氧酪胺在BEH C18上保留较弱,基质加标样品目标峰处有明显杂质干扰峰,通过改变有机相种类和梯度无明显变化,考虑到出峰时间太早不利于目标峰和杂质峰的分离,故更换对极性化合物保留效果更好且耐纯水相的HSS T3色谱柱。在相同梯度下分别以甲醇和乙腈作有机相,发现乙腈作有机相时大部分化合物响应较甲醇作有机相时降低30%~50%,仅出峰较晚的两个化合物响应略有增加,故选用甲醇做流动相。实际样品测定中发现,出峰较早的甲氧酪胺和阿替洛尔目标峰处干扰较大,可能是由于净化步骤去除的更多为非极性物质,而部分未能被水除去的极性较强的杂质与出峰较早的目标物共同流出,通过降低有机相初始比例来延长较早出峰的化合物保留时间,以保证不同基质样品中目标峰与杂质的很好分离。在色谱柱和流动相梯度确定后,进一步比较了不同定容液对各化合物的影响,实验比较了0.1%甲酸水、乙腈-0.1%甲酸水(1∶9, v/v)、乙腈-0.1%甲酸水(2∶8, v/v)、乙腈-0.1%甲酸水(3∶7, v/v)4种定容液,发现甲氧酪胺在乙腈-0.1%甲酸水(2∶8, v/v)、齐帕特罗、阿替洛尔在乙腈-0.1%甲酸水(3∶7, v/v)时峰形分叉为双峰,说明随着有机相比例增大,溶剂效应明显;而出峰较晚的脱氢异雄酮、美替诺龙随着有机相比例增大响应明显提高,综合考虑选用乙腈-0.1%甲酸水(1∶9, v/v)作为定容液。2.2.2 质谱条件通过优化离子对和碰撞能,获得MRM采集参数,选择响应值较高的子离子作为定量离子,另一个作为定性离子。分段扫描以增大驻留时间提高响应,同时开启质谱和废液切换阀,以降低仪器维护频率。

### 2.3 基质效应

基质效应是指色谱分离时基质中存在的干扰物与待测目标化合物共洗脱从而改变待测组分的离子化效率,引起待测组分信号提高或抑制。选取阴性猪肉、鸡蛋、牛奶作为基质,经前处理后作为空白基质溶液与溶剂分别配制相同浓度的点进样分析。以空白基质标准点与溶剂标准点峰面积相比增加或减少的百分比来衡量基质效应,即ME=(基质空白标准点峰面积/溶剂标准点峰面积-1)×100%表示基质效应,当ME>0时为基质增强,ME<0时为基质抑制,ME的绝对值<20%认为不存在明显的基质效应。

**图 3:**
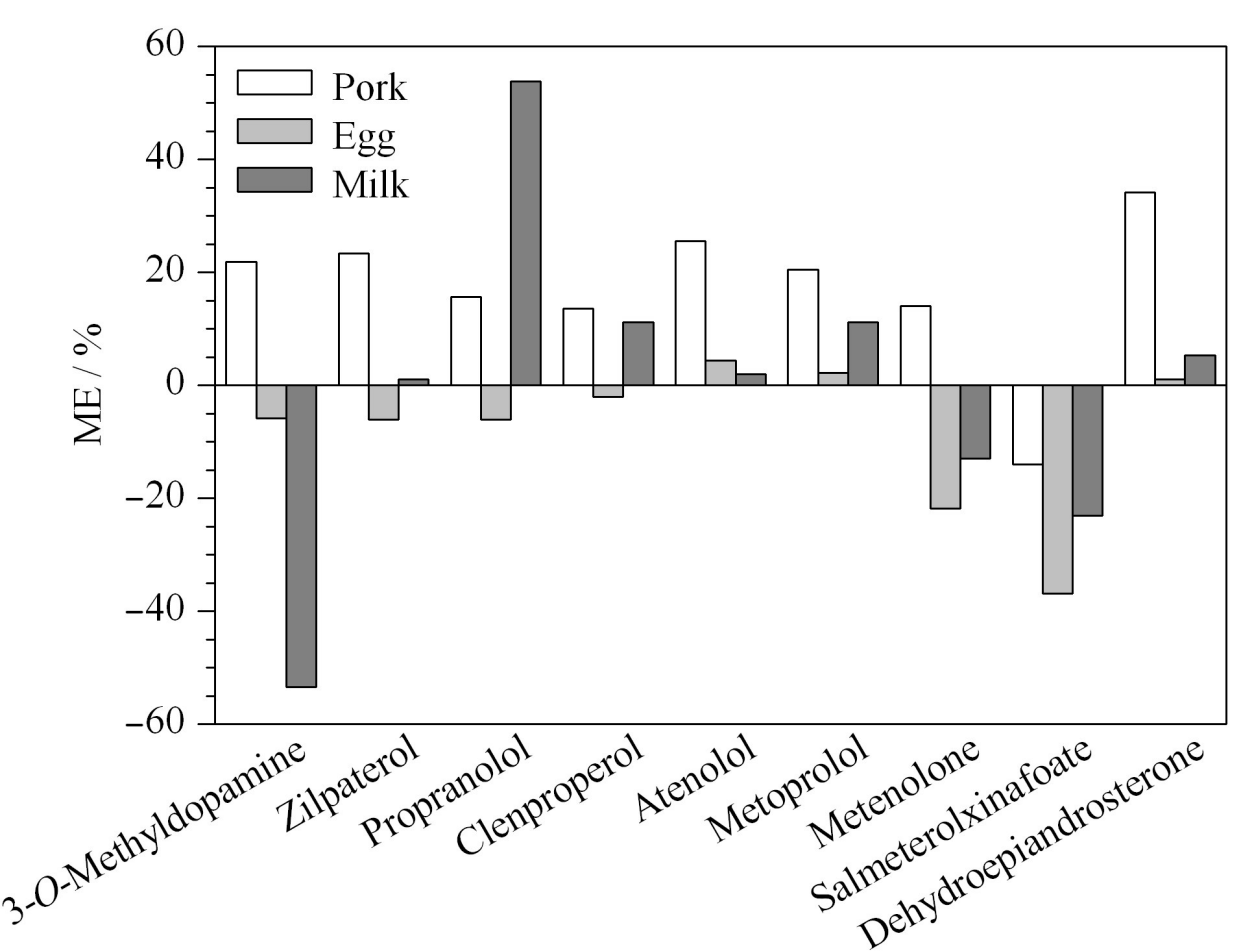
不同基质中9种兴奋剂的基质效应

in different matrices结果表明,不同基质中的基质效应各有不同,见图3,猪肉中除齐帕特罗、阿替洛尔、脱氢异雄酮为基质增强外,其他化合物均不存在明显的基质效应;鸡蛋中美替诺龙、沙美特罗存在基质抑制效应,牛奶中甲氧酪胺和普萘洛尔分别存在明显的基质抑制和基质增强。故实验采用空白基质标准曲线,由于未能获得所有化合物的内标,结合回收率、化合物结构和出峰时间选取合适的内标定量,获得良好的结果。

### 2.4 检出限、定量限与线性范围

选取猪肉、鸡蛋、牛奶3种空白基质,配制不同浓度的基质标准溶液,经液相色谱-串联质谱检测,以3倍信噪比为检出限,10倍信噪比为定量限,方法检出限(LOD)为0.3~0.6 μg/kg,定量限(LOQ)为1.0~2.0 μg/kg;以待测物与相对应同位素内标的峰面积之比为纵坐标(*y*),相应的质量浓度为横坐标(*x*, μg/L),绘制标准曲线。结果如表2所示,3种基质的基质匹配曲线9种待测物在各自范围内线性关系良好,相关系数(*r*^2^)大于0.99。

**表 2:** 9种食源性兴奋剂类药物的线性范围、线性方程、相关系数、检出限和定量限

Compound	Linear range/(μg/L)	LOD/(μg/kg)	LOQ/(μg/kg)	Matrix	Linear equation	r^2^	
3-O-Methyldopamine	0.5-20	0.3	1.0	pork	y=0.0151109x+0.00214252	0.9972	
				egg	y=0.00603772x+0.00149372	0.9966	
				milk	y=0.012718x+0.00247736	0.9993	
Zilpaterol	0.5-20	0.3	1.0	pork	y=0.206449x+0.0151934	0.9986	
				egg	y=0.138101x+0.00342916	0.9995	
				milk	y=0.211776x+0.0164704	0.9960	
Propranolol	0.5-20	0.3	1.0	pork	*y*=0.0730639*x*+0.0038951	0.9987
				egg	*y*=0.180135*x*+0.00280754	0.9986
				milk	*y*=0.0739471*x*+0.00527138	0.9982
Clenproperol	0.5-20	0.3	1.0	pork	*y*=0.0386475*x*+0.00076196	0.9989
				egg	*y*=0.118687*x*+0.000713237	0.9970
				milk	*y*=0.035279*x*+0.00118618	0.9990
Atenolol	0.5-20	0.3	1.0	pork	*y*=0.0622948*x*+0.00392713	0.9986
				egg	*y*=0.130383*x*-0.000330244	0.9994
				milk	*y*=0.0616749*x*+0.00596514	0.9971
Metoprolol	0.5-20	0.3	1.0	pork	*y*=0.172068*x*+0.0133167	0.9983
				egg	*y*=0.542915*x*+0.00151761	0.9999
				milk	*y*=0.174471*x*+0.0174125	0.9973
Metenolone	0.5-20	0.3	1.0	pork	*y*=0.108278*x*+0.0205757	0.9999
				egg	*y*=0.168793*x*-0.00348651	0.9992
				milk	*y*=0.109783*x*-0.00169571	0.9996
Salmeterolxinafoate	0.5-20	0.3	1.0	pork	*y*=0.183648*x*+0.0116977	0.9987
				egg	*y*=0.521194*x*+0.020523	0.9993
				milk	*y*=0.176511*x*+0.0131412	0.9982
Dehydroepiandrosterone	1-40	0.6	2.0	pork	*y*=0.0138347*x*+0.0166979	0.9975
				egg	*y*=0.00661188*x*+0.0141375	0.9954
				milk	*y*=0.0134266*x*+0.0211796	0.9941

*y*: ratio of the peak areas of compound to internal standard; *x*: mass concentration, μg/L.

### 2.5 加标回收率与精密度

选取空白猪肉、鸡蛋、牛奶样品,分别添加低、中、高3个不同浓度水平的混合标准溶液,按1.3节进行前处理,每个水平重复测定6次,基质匹配曲线内标法定量,计算回收率和相对标准偏差(RSD)。如表3所示,9种目标化合物的平均回收率为65.2%~117.0%, RSD为1.3%~14.4%。

**表 3:** 猪肉、鸡蛋、牛奶样品中9种食源性兴奋剂类药物的回收率和精密度(*n*=6)

Compound	Spiked level/ (μg/kg)	Recoveries/%				RSDs/%		
		Pork	Egg	Milk		Pork	Egg	Milk
3-*O*-Methyldopamine	1	85.5	89.3	72.2		10.7	6.0	10.1
	2	79.3	84.8	69.2		10.8	4.4	4.4
	5	71.7	75.7	65.5		8.3	3.9	2.9
Zilpaterol	1	89.0	83.8	76.7		9.7	5.1	5.1
	2	80.0	79.3	80.7		13.9	4.7	6.3
	5	76.0	71.8	68.7		14.3	5.9	2.9
Propranolol	1	117.0	111.0	113.0		1.6	3.4	4.7
	2	110.0	110.0	111.0		8.3	3.3	5.5
	5	108.0	110.0	113.0		3.5	3.2	7.8
Clenproperol	1	113.0	111.0	116.0		3.7	1.7	3.9
	2	108.0	112.0	114.0		7.3	3.1	3.2
	5	101.0	107.0	115.0		1.3	2.0	1.7
Atenolol	1	82.0	78.7	69.2		14.0	4.2	10.7
	2	75.9	86.7	76.0		14.4	12.5	5.8
	5	72.7	89.6	69.2		13.9	5.0	1.3
Metoprolol	1	117.0	115.0	115.0		1.7	2.7	4.6
	2	116.0	112.0	113.0		2.1	4.1	4.4
	5	113.0	111.0	108.0		3.2	3.5	7.7
Metenolone	1	75.4	70.3	78.0		10.0	3.8	13.8
	2	72.7	92.4	71.6		11.5	8.4	4.0
	5	65.2	75.8	66.3		2.5	8.0	3.6
Salmeterolxinafoate	1	73.9	80.7	83.5		7.2	3.4	12.5
	2	72.0	65.4	80.4		6.7	5.1	6.2
	5	70.9	73.9	87.2		4.4	7.2	11.7
Dehydroepiandrosterone	2	74.3	106.0	73.7		13.6	4.3	8.3
	4	81.0	103.0	83.1		13.8	5.0	8.3
	10	82.7	86.8	85.1		7.0	8.0	5.4

### 2.6 实际样品检测

测定了市售的猪肉、鸡蛋、牛奶样品共计50批次,均未有检出。

## 3 结论

建立了一种基于QuEChERS EMR Lipid净化结合超高效液相色谱-串联质谱测定猪肉、鸡蛋、牛奶中9种兴奋剂类药物残留的方法。该方法前处理简单,溶剂用量少,对脂质含量较高的动物源性食品净化效果好,适用于大批量样品的快速、准确测定,可为多种食源性兴奋剂的快速检测技术研究提供参考。
